# Wavelength-Tuneable Near-Infrared Luminescence in Mixed Tin–Lead Halide Perovskites

**DOI:** 10.3389/fchem.2022.887983

**Published:** 2022-05-31

**Authors:** Meiyue Liu, Ru Zhao, Fuhao Sun, Putao Zhang, Rui Zhang, Zeng Chen, Shengjun Li

**Affiliations:** Key Laboratory of Photovoltaic Materials, Henan University, Kaifeng, China

**Keywords:** Near-Infrared luminescence, mixed tin-lead halide perovskite, optical properties, wavelength-tuneable, stability

## Abstract

Near-infrared light-emitting diodes (NIR-LEDs) are widely used in various applications such as night-vision devices, optical communication, biological imaging and optical diagnosis. The current solution-processed high-efficiency perovskite NIR-LEDs are typically based on CsPbI_3_ and FAPbI_3_ with emission peaks being limited in the range of 700–800 nm. NIR-LEDs with longer emission wavelengths near to 900 nm can be prepared by replacing Pb with Sn. However, Sn-based perovskite LEDs usually exhibit a low efficiency owing to the high concentration of Sn-related defects and the rapid oxidation of Sn^2+^ to Sn^4+^, which further induces the device degradation. These problems can be solved by rationally adjusting the ratio between Pb content with Sn. Mixed Sn-Pb halide perovskites with a smaller bandgap and superior stability than pure Sn-based perovskites are promising candidates for manufacturing next-generation NIR emitters. In this study, we systematically investigated the optical properties of a family of hybrid Sn and Pb iodide compounds. The emission spectra of the mixed Sn-Pb halide perovskites were tuned by changing the Sn:Pb ratio. Consequently, the peak emission wavelength red-shifted from 710 nm to longer than 950 nm. The absorption and photoluminescence emission properties associated with different compositions were compared, and the results demonstrated the potential of MA- and FA-based mixed Sn-Pb halide perovskites for preparing low-cost and efficient NIR-LEDs. In addition, we clarified the influence of cations on the bandgap bowing effect and electronic properties of mixed Sn-Pb halide perovskites.

## Introduction

The near-infrared (NIR) spectrum refers to electromagnetic waves with wavelengths ranging from 700 to 2500 nm. NIR light is invisible, which has deep-depth tissue penetration and is less hazardous to living organisms. Light-emitting diodes (LEDs) with emissions in the NIR region (termed NIR-LEDs) can be used in a wide variety of applications such as biological imaging, night-vision devices, optical communication, remote sensing and optical diagnosis (Tessler et al., 2002; Smith et al., 2009; Xiang et al., 2013; Gu et al., 2019). Current NIR-LEDs are typically prepared using III-V inorganic semiconductors that are epitaxially grown on crystalline substrates (Kato et al., 1991; Saka et al., 1993; Dimakis et al., 2014; Zhong and Dai, 2020). However, the processing of III-V LEDs, that requires high vacuum and high-temperature sintering treatments, increases the manufacturing costs (Kato et al., 1991; Saka et al., 1993). Therefore, organic LEDs and colloidal quantum dot (QD) LEDs have been developed, which can be processed using low-cost and low-temperature methods. High-efficiency organic and colloidal quantum dot LEDs with tuneable emission wavelengths have been reported (Borek et al., 2007; Hinds et al., 2007; Supran et al., 2015; Kim et al., 2018). Although organic LEDs can be fabricated in a facile manner, the synthesis of organic materials with emissions in the infrared range involves complex processes. Moreover, such LEDs exhibit low thermal stability and luminance (Sandanayaka et al., 2015). Colloidal QD LEDs offer several advantages such as high brightness, high efficiency and solution-processing compatibility. However, the use of cadmium (Cd) and lead (Pb) is harmful to the environment, and the synthesis of QDs requires high-temperature reactions to be performed over long periods (Shirasaki et al., 2013).

Metal halide perovskites exhibit excellent optoelectronic properties such as long charge carrier diffusion length, bandgap tuneability, high defect tolerance and high photoluminescence quantum yield and can thus be applied in several optoelectronic devices (Sutherland and Sargent, 2016; Song et al., 2019). The general formula of perovskites is ABX_3_, where A is an organic cation (MA, FA, or Cs), B is a divalent metal (Pb^2+^ or Sn^2+^) and X is a halide anion (I^−^, Br^−^ or Cl^−^). The most recently reported power conversion efficiency (PCE) of Pb-based perovskite solar cells has reached 25.7% (NREL, 2022). LEDs with emission wavelengths ranging from 400 to 800 nm have been demonstrated based on Pb-based perovskites. The peak external quantum efficiency (EQE) of LEDs has undergone a rapid increase from lower than 1% in 2014 to higher than 20% in 2021 (Deschler et al., 2014; Cao et al., 2018; Chiba et al., 2018; Lin et al., 2018; Li et al., 2019; Wang et al., 2019; Chen et al., 2021; Liu et al., 2021). However, the emission wavelength of Pb-based perovskites is tuneable only to near 800 nm, which limits their NIR applications. Narrow-bandgap materials must be used to obtain longer wavelength emissions. Moreover, the toxicity of Pb hinders the commercialisation of perovskite LEDs (Liu et al., 2018; Zhu et al., 2020). Replacing all or part of the Pb content with tin (Sn) can alleviate the problems associated with the toxicity of Pb (Wang et al., 2017; Zhang et al., 2018; Lin JT. et al., 2019; Jiang et al., 2020; Wang et al., 2020). The replacement of Pb with Sn can also obtain smaller bandgaps than those of Pb-based perovskites, resulting in longer wavelength emissions (Hao et al., 2014; Lai et al., 2016).

Sn-based perovskites with long emission wavelengths have emerged as promising candidates for preparing NIR-LEDs. In 2016, Lai et al. demonstrated tuneable NIR electroluminescence in Sn halide perovskites by adjusting the halide content of I^−^ and Br^−^. The authors achieved a 945 nm emission with a radiance of 3.4 W sr^−1^ m^−2^ and maximum EQE of 0.72% (Lai et al., 2016). For high device performance, a uniform perovskite film must be needed. Hong et al. prepared all-inorganic CsSnI_3_ films *via* the toluene dripping method and observed the presence of compact micrometre-sized grains with extremely few pinholes or cracks at the grain boundaries. The NIR-LEDs with the CsSnI_3_ film exhibited an emission peak at 950 nm with a maximum radiance of 40 W sr^−1^ m^−2^ (Hong et al., 2016). Since these studies, the EQE has been gradually increased, but device advancements have proven challenging. In 2021, Lu et al. used a dendritic structure to develop an efficient CsSnI_3_-based perovskite NIR-LED. They found unbalanced charge injection, that the hole injection is higher than that of electron, was a cause of poor device performance. The dendritic structure remedied this by increasing electron injection, and the CsSnI_3_-based perovskite LEDs based on this structure obtained a record EQE of 5.4% with an acceptable efficiency roll-off and a high radiance of 162 W sr^−1^ m^−2^ (Lu et al., 2021). However, the efficiency of Sn-based NIR-LEDs remains considerably lower than that of Pb-based devices owing to the rapid oxidation of Sn^2+^ to Sn^4+^, which leads to poor device performance and accelerates degradation. Although the complete replacement of Pb with Sn seems challenging at present, this issue was expected to be solved by partially replacing Pb^2+^ with Sn^2+^.

Mixed Sn-Pb perovskites, which exhibit a small bandgap around 1.2 eV, have been used to construct high-efficiency low-bandgap perovskite solar cells and all-perovskite tandem devices (Eperon et al., 2016; Lin et al., 2022; Yu et al., 2022). Moreover, mixed Sn-Pb halide perovskites possess superior stability, as compared to pure Sn perovskites (Leijtens et al., 2017; Lin R. et al., 2019; Yang et al., 2019). Recently, Qiu et al. tuned the NIR spectral region from 800 to 950 nm by changing the ratio of Pb^2+^ and Sn^2+^. Furthermore, the authors showed that the addition of 4-fluorobenzylammonium iodide (FPMAI) yielded the best-performing device achieving a maximum EQE of 5% with an emission peak at 917 nm (Qiu et al., 2019). In general, photoluminescence (PL) at 900–1000 nm is suitable for biomedical imaging, information communication and wound treatment. Although the performance of mixed Sn-Pb perovskite devices with longer wavelength emissions is inferior to that of Pb-based perovskites, the tuneable wavelength emission and NIR up to wavelengths of ∼1000 nm is very attractive for perovskite NIR-LEDs.

In this study, we systematically investigated the properties of MASn_x_Pb_1-x_I_3_ (MA), FASn_x_Pb_1-x_I_3_ (FA) and CsSn_x_Pb_1-x_I_3_ (Cs) (x = 0, 0.2, 0.4, 0.6, 0.8 and 1), focusing on their optical, structural, morphology and electronic properties. Ultraviolet–visible (UV–vis) absorption, PL emission and PL lifetime were measured to study the optical properties, and X-ray diffraction (XRD) and scanning electron microscopy (SEM) analyses were performed for structural and morphological characterisation. The ultraviolet photoelectron spectroscopy (UPS) was performed for electronic properties. The results demonstrated that the emission wavelength could be tuned from 710 nm to approximately 950 nm by changing the Sn:Pb ratio. The structure, morphology and optical properties associated with different compositions were compared, and the results indicated that the MA- and FA-based mixed Sn-Pb perovskites are promising candidates for manufacturing next-generation NIR emitters with wavelengths exceeding 900 nm and high spectral tuneability. Moreover, from the results of bandgap values of these perovskites compositions, we clarified the influence of cations on the bandgap bowing and electronic properties of mixed Sn-Pb halide perovskites.

## Results and Discussion

All of the perovskite films were prepared by solution processing, and the mixed Sn-Pb halide perovskite solutions were prepared by mixing pure Pb-based and Sn-based perovskite solutions in stoichiometric ratios (details are provided in the Experimental Section). To investigate the effect of the Sn:Pb ratio on the optical properties, we performed various characterisations and measurements, including UV–vis absorption, PL emission and PL lifetime measurements. Notably, the characterisations were performed in the ambient environment, and Sn-based perovskites, especially all-inorganic CsSnI_3_ perovskites, are easily degraded in air. Therefore, a small amount of phenylammonium iodide (PEAI) was added to the Cs-based perovskite precursor solutions to enhance their stability. To maintain uniformity, an equal amount of PEAI was also added to the precursor solutions of the FA- and MA-based perovskites. PEAI is widely used in perovskite solar cells to improve device stability, usually in one of two ways: 1) PEAI can replace a certain fraction of MA, FA and Cs cations to form a protecting layer with a two-dimensional (2D) or 2D/3D perovskite structure. 2) Alternatively, a small amount of PEAI can be added into the standard precursor solution so that it does not form 2D perovskites but binds to the crystal surface as capping ligands to stabilize the existing perovskite structure (Fu et al., 2017; Kuo et al., 2021; Zhang et al., 2021). In this work, to maintain the 3D structure of the films, we followed the practice of 2) and added only a small amount of PEAI into the standard precursor solutions by volume.

**FIGURE 1:**
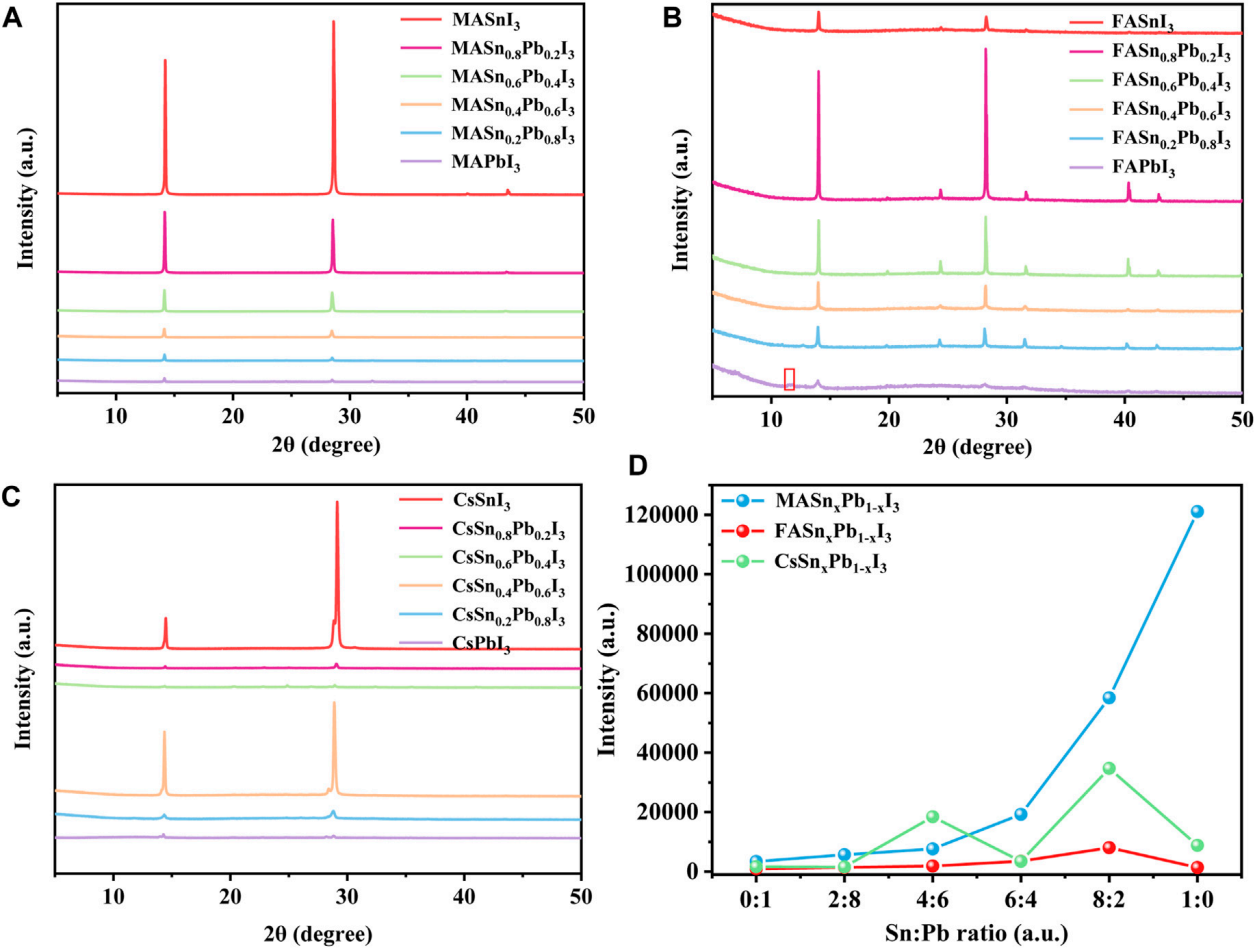
XRD patterns of **(A)** MASn_x_Pb_1-x_I_3_, **(B)** FASn_x_Pb_1-x_I_3_ and **(C)** CsSn_x_Pb_1-x_I_3_; **(D)** (110) peak intensity for each composition.

Specifically, first, we added a 50 μL PEAI solution with a concentration of 100 mg/ml to the standard perovskite precursor solutions. XRD measurements were performed to examine whether the addition of PEAI changed the structure of the perovskites. The XRD patterns of the reference films are shown in Supplementary Figure S1. The peak at 2θ = 11.7° is correlated to δ-FAPbI_3_ (yellow phase) in the FAPbI_3_ film, while the peak at 2θ = 13.9° represents the α-FAPbI_3_ (black phase). The XRD patterns of films with added PEAI are shown in Supplementary Figure S2. The peak at 11.7° is weak in the FAPbI_3_ film deposited with the presence of PEAI. While a new peak can be observed at a small 2θ angle of approximately 7.1°, indicating the formation of the 2D perovskite structure. To maintain the 3D structure, we next decreased the PEAI concentration to 50 mg/ml. Figure 1 shows the XRD patterns of the resulting perovskite films. No new peaks were observed, which indicated that this amount of PEAI did not change the structure of the perovskites. Figure 1A shows the XRD patterns of the MASn_x_Pb_1-x_I_3_ perovskites. The XRD intensity increased with the increase in the Sn proportion. Consequently, the MASnI_3_ exhibiting the highest intensity. This demonstrated that the Sn-based perovskites exhibited better crystallisation than the Pb-based perovskites. A similar trend was observed for the FA-based perovskites, as shown in Figure 1B, wherein the XRD intensity increased as the Sn proportion increased. Interestingly, the FASnI_3_ did not exhibit the highest intensity, possibly because of the inferior film morphology featuring large pinholes as compared to the mixed Sn-Pb perovskites. In the case of the Cs-based perovskites, the intensities of the mixed Sn-Pb halide and pure Sn perovskites were higher than those of the pure Pb perovskite, although a clear rule could not be identified (Figure 1C). To examine the effect of the Sn:Pb ratio on the crystallinity, we compared the (110) peak intensities of different compositions (Figure 1D). The results showed that the intensity increased with the increase in the Sn:Pb ratio, and the highest intensity was observed when Sn:Pb ratio is 8:2 (except in the case of MASnI_3_). This behaviour was attributable to two factors. First, the Sn:Pb ratio may have changed the tolerance factor, thereby affecting the XRD intensity, as reported in a previous study (Kieslich et al., 2015). We calculated the tolerance factor of each composition and found that with the increase of Sn ratio, the values get closer to 1(Supplementary Figure S3 and Supplementary Table S1). Second, the complex morphology, especially the film coverage, may have affected the XRD intensity. The SEM images showed a varied morphology for the mixed Sn-Pb halide perovskites while the pure Sn-based perovskite films had large pinholes in MA- and Cs-based perovskites. In contrast, the morphology of the FA-based mixed Sn-Pb halide perovskites was relatively smooth, and the grains were uniform (Supplementary Figure S4).

Among the MA-, FA- and Cs-based perovskites, the MA-based perovskites exhibited the highest XRD intensity, corresponding to the highest crystallinity. Moreover, compared with those of the pure Pb-based perovskites, the XRD peaks of the mixed Sn-Pb halide and pure Sn-based perovskites shifted slightly towards larger angles, indicating the incorporation of Sn^2+^, which has a smaller ionic radius than Pb^2+^ (Supplementary Figure S5). The relationships between the angle shift of the XRD peak and the Sn:Pb ratio in the three perovskite systems are presented in Supplementary Figure S5. The FA- and Cs-based perovskites exhibited the smallest and largest shifts in angle, respectively, indicating that the Sn:Pb ratio most notably influenced the lattice in Cs-based perovskites.

**FIGURE 2:**
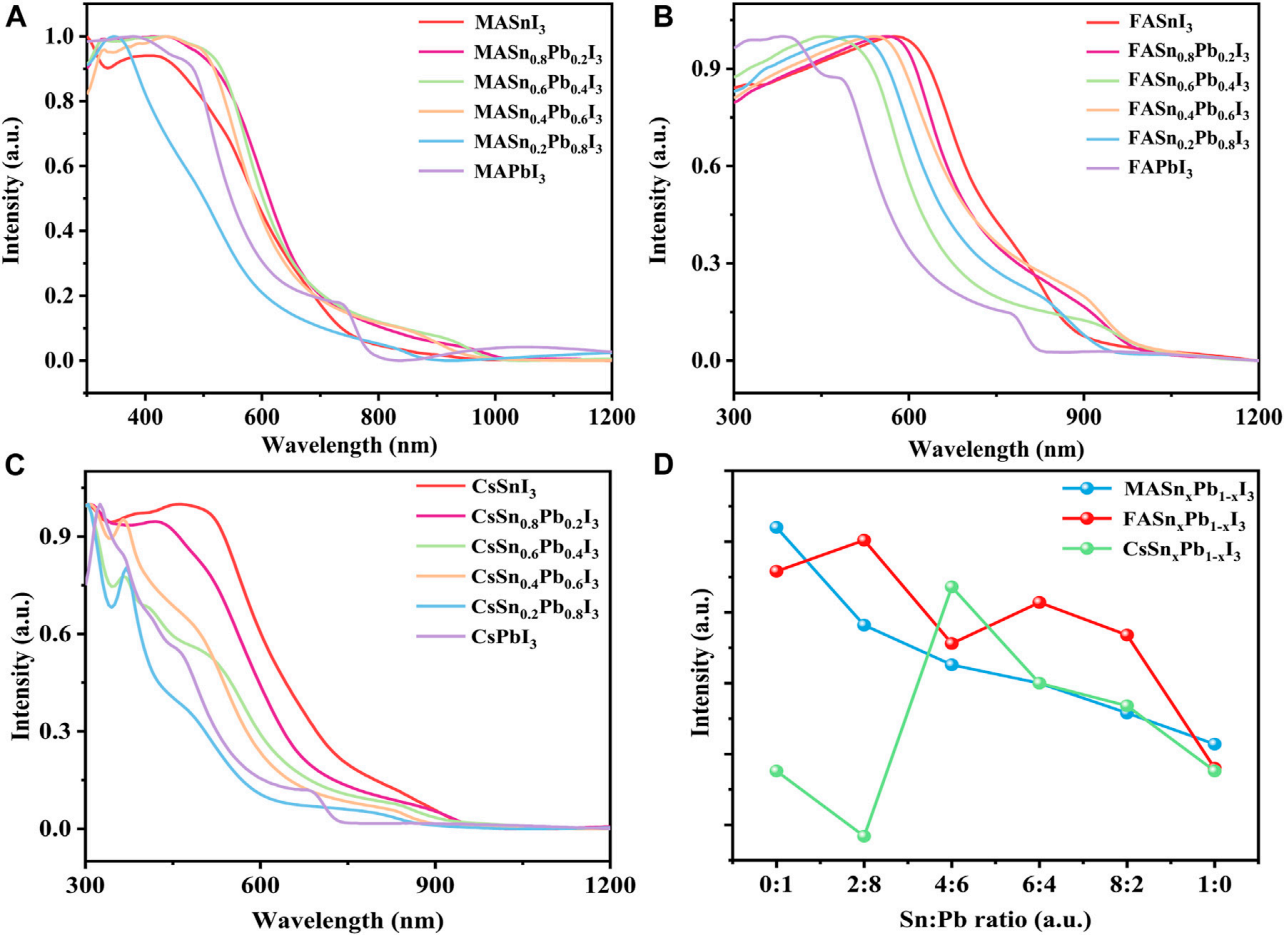
Optical absorption properties of **(A)** MASn_x_Pb_1-x_I_3_, **(B)** FASn_x_Pb_1-x_I_3_ and **(C)** CsSn_x_Pb_1-x_I_3_; **(D)** absorption intensity for each composition.

The absorption spectra of mixed Sn-Pb halide perovskites can be tuned by changing the Sn-Pb stoichiometry (Lim et al., 2021; Savill et al., 2021). Figure 2 shows the absorption spectra of perovskites with different Sn:Pb ratios. All materials exhibited strong absorption in the visible spectral region, and in the mixed Sn-Pb halide perovskites the absorption region extended to ∼950 nm. The absorption region of the MA- and FA-based perovskites did not follow a linear trend as a function of x, in contrast to the case of the Cs-based perovskites. For example, Figure 2A shows that for the MA-based perovskites, the absorption edge initially red-shifted from approximately 800–1000 nm as the Sn content increased, followed by a blue-shift when the Sn-Pb ratio further increase to 8:2 or even pure Sn perovskite. Similar trend was observed for the FA-based perovskite systems, as shown in Figure 2B. These results are consistent with those reported previously (Hao et al., 2014; Goyal et al., 2018). In contrast to the MA- and FA-based perovskites, the Cs-based perovskites did not exhibit a blue-shift at the high Sn-Pb ratio (Figure 2C), probably because of the absence of organic cations (Hu et al., 2020; Xia et al., 2020), as discussed in the following paragraph. In addition, we compared the absorption intensity of each composition, as shown in Figure 2D. All materials exhibited relatively high absorption intensity, although the intensity decreased as the Sn ratio increased. The pure Sn-based perovskites thus exhibited the lowest absorption intensity, probably because of the high concentration of Sn-related defects and poor film morphology owing to the fast crystallisation of Sn-based perovskites.

**FIGURE 3:**
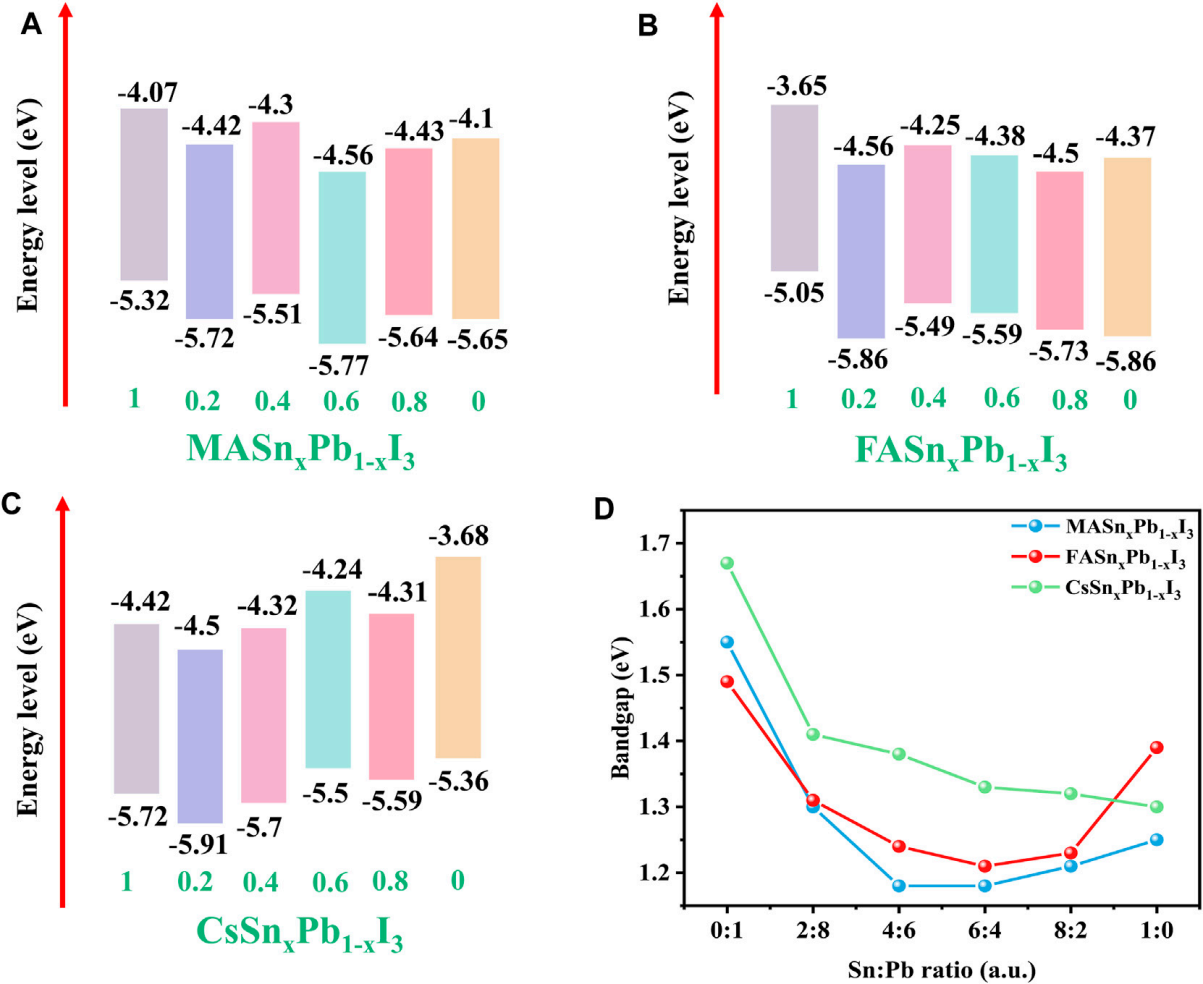
Energy levels of **(A)** MASn_x_Pb_1-x_I_3_, **(B)** FASn_x_Pb_1-x_I_3_ and **(C)** CsSn_x_Pb_1-x_I_3_ where x = 1, 0.2, 0.4, 0.6, 0.8, 0; **(D)** relationship between bandgap and Sn:Pb ratio.

To investigate the influence of the Sn:Pb ratio on the energy levels of the mixed Sn-Pb halide perovskites, UPS analysis was performed to derive the secondary electron cut-off and valence band (VB) edge of the perovskites. Using these results and the absorption spectra, we calculated the conduction band (CB). The energy levels of MA-, FA- and Cs-based perovskites are shown in Figures 3A–C, respectively. The UPS spectra are shown in Supplementary Figure S6. The bandgaps for each composition are summarised in Figure 3D. Compared with the Cs-based perovskites, the MA- and FA-based perovskites exhibited an obvious bowing effect, and the lowest bandgap was achieved when the Sn:Pb ratio is 4:6 or 6:4.

The nonlinear bandgap behaviour of mixed Sn-Pb halide perovskites is a consequence of chemical effects and the mismatch energy between the s and p atomic orbitals of Pb and Sn. The Sn-s and Sn-p atomic orbitals are less strongly bound than those in the corresponding Pb state. Consequently, the VB maximum in the alloy is derived from interactions between the Sn-s and I-p orbitals, and the CB minimum is derived from the Pb-p and I-p orbitals. As a result, the bandgap is smaller than that of either end compound (Goyal et al., 2018). While the Cs-based perovskite did not exhibit this behaviour, with the observed trend being nearly linear, the bandgap decreased as the Sn content increased and CsSnI_3_ exhibited lowest bandgap. These findings are consistent with those reported previously, in which the absence of this trend was attributed to the absence of organic cations (Hu et al., 2020; Xia et al., 2020). Xia et al. investigated the electronic properties of all-inorganic perovskite CsSn_1-x_Pb_x_Br_3_ and MA_y_Cs_1-y_Sn_1-x_Pb_x_Br_3_ materials. The authors demonstrated that the Sn and Pb atoms occupied distinct sites in the two mixed compounds, and the bandgap bowing parameter of CsSn_1-x_Pb_x_Br_3_ was the smallest among all the considered perovskite materials. This result indicated the influence of cations on bandgap bowing effect (Xia et al., 2020). Comparing the maximum and minimum bandgaps for the three perovskite systems showed that the bandgap for the Cs-based perovskites exhibited the largest change, from approximate 1.7 eV to near 1.3 eV, indicating that bandgap tuning is easier to achieve with Cs-based perovskites. This result is consistent with those derived from the XRD patterns. The largest angle shift was observed for Cs-based perovskites, which indicates that Sn^2+^ replacement influences the lattice structure for these perovskites more significantly than for the MA- and FA-based perovskites (Hee et al., 2019; Kim et al., 2019).

**FIGURE 4:**
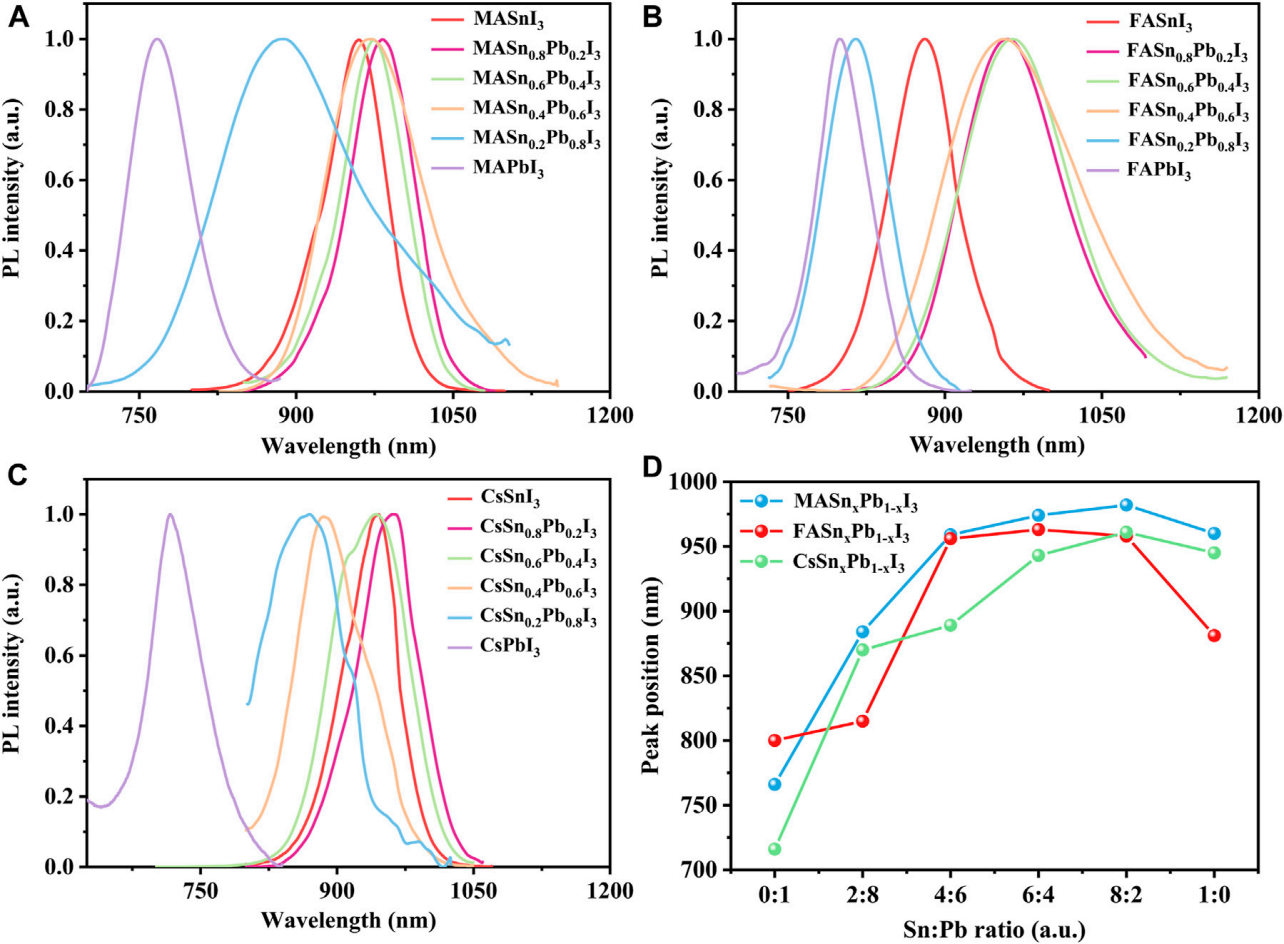
Normalised PL spectra of **(A)** MASn_x_Pb_1-x_I_3_, **(B)** FASn_x_Pb_1-x_I_3_ and **(C)** CsSn_x_Pb_1-x_I_3_; **(D)** PL peak position of each composition.

The PL properties of all of the perovskites were measured at room temperature by using a green light emission source with a wavelength of 532 nm. The Sn and Pb compounds exhibited intense PL emissions. The Pb analogues exhibited a peak emission wavelength in the 700–800 nm range, and the mixed Sn-Pb and Sn analogues exhibited emissions at longer wavelengths, between 850 nm and around 1000 nm, covering a broad region of the NIR spectrum (Figure 4). An obvious shift in the peak emission wavelength was observed, consistent with the corresponding absorption energy edge. For example, in the case of the MA-based perovskites, MAPbI_3_ exhibited the peak emission at 766 nm. However, as the addition of Sn (x = 0.2, 0.4, 0.6, 0.8), the emission maximum red-shifted to 860 nm, 969 nm, 974 nm and 982 nm, respectively, followed by a blue-shifted emission to 960 nm when further increasing Sn proportions to the pure Sn perovskite, i.e., MASnI_3_ (Figure 4A). This result is consistent with the anomalous bandgap that was reported for mixed Sn-Pb halide perovskites (Hao et al., 2014). The PL emission spectra of the FA- and Cs-based perovskites are shown in Figures 4B,C, the peak position of each composition is plotted in Figure 4D, and the peak position data are summarised in Supplementary Table S2. The reddest emission was achieved by MASn_0.8_Pb_0.2_I_3_, with a wavelength at 982 nm in the NIR. The characterization results showed that Sn:Pb ratio of 8:2 is the turning point for the most properties of perovskites, which is suspected to be related to tolerance factor. As shown in Supplementary Figure S3, the tolerance factor for the Sn:Pb ratio 8:2 approached 1, similar to that of the pure Sn-based perovskites.

Similar to the results for the absorption spectra, the Cs-based mixed perovskites covered a wide PL emission range from 716 to 960 nm. In addition to the variation in peak shift, the full-width half-maximum of the PL spectra were considerably different, as shown in Figures 4A,B, attributable to the poor, non-uniform morphology of the films, as shown in Supplementary Figure S4. Park et al. reported that the film microstructure (i.e., grain size, roughness and presence of defects) can influence the PL properties (Park et al., 2018). The PL intensity of each composition is shown in Supplementary Figure S7. The Cs-based perovskites exhibited the weakest PL intensity, attributable to either the instability of Cs-based perovskites or the poor morphology of the films, as observed in the SEM images.

Compared with the absorption spectra, the PL spectra exhibited an obvious blue shift, caused by the Stark effect (Roiati et al., 2014; Pazoki et al., 2017). The Stark shifts calculated from the absorption and PL spectra are shown in Supplementary Figure S8. The Cs-based perovskites exhibited a small Stark shift of approximately 10 nm, whereas the FA- and MA-based perovskites exhibited a large Stark shift of about 50 nm. Typically, the Stark shift is associated with photon reabsorption, and a large Stark shift indicates weak reabsorption. Therefore, the findings demonstrate the strong reabsorption capacities of the Cs-based perovskites.

**FIGURE 5:**
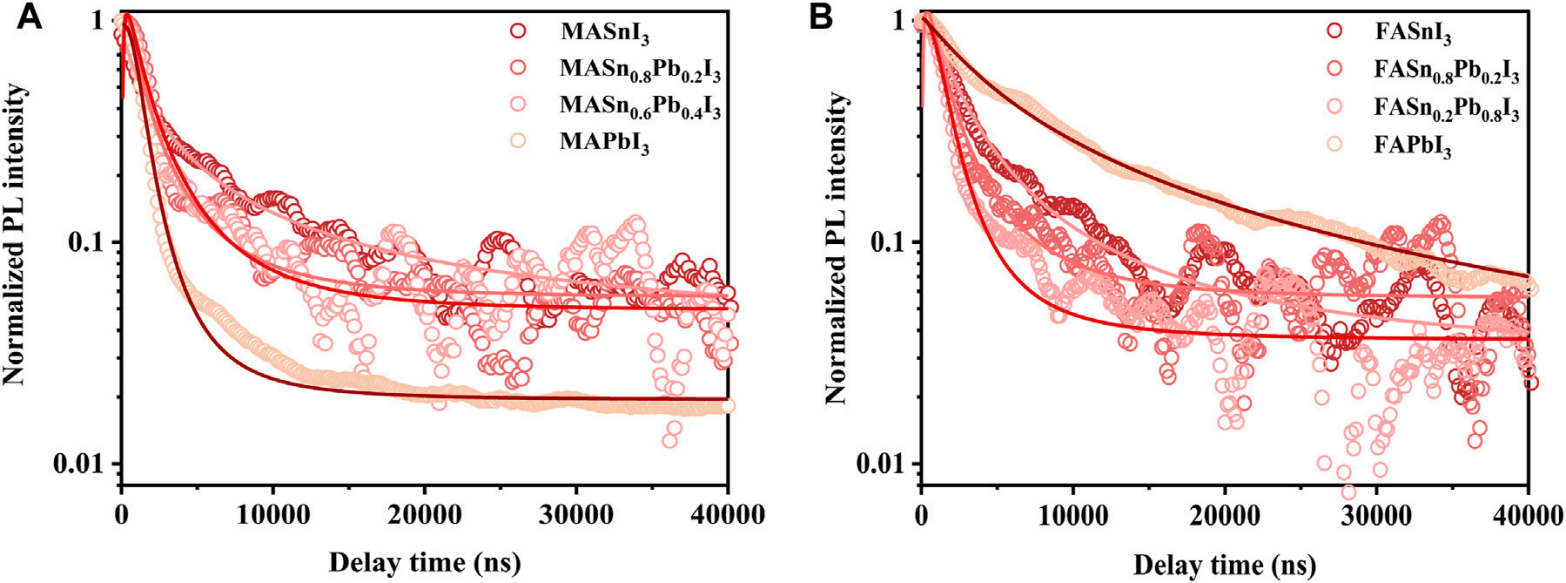
PL decay lifetime of perovskites: **(A)** MASn_x_Pb_1-x_I_3_, **(B)** FASn_x_Pb_1-x_I_3_.

Figure 5 shows the PL lifetimes of the perovskite films, acquired using a laser as the excitation source with a central wavelength at 532 nm and a repetition rate of 1 kHz. Figure 5A shows the PL lifetimes of the MA-based perovskite films with different Sn:Pb ratios. The MASnI_3_ perovskites exhibited the longest lifetimes. In the case of the mixed Sn-Pb halide perovskites, as the Sn:Pb ratio increased, the PL lifetime continued to increase, and the Sn:Pb ratio of 8:2 corresponded to longer lifetime. Similar trend was observed for the FA-based perovskites (Figure 5B). The PL lifetime of the FA-based perovskites was longer than that of the MA-based perovskites, indicating slower recombination. This result is attributable to the higher formation energy of Sn vacancy for FASnI_3_ perovskites compared with that of MASnI_3_ perovskites (Shi et al., 2017). As shown in Figure 5, certain PL lifetime data could not be obtained owing to weak signals, as observed for the PL intensity without normalisation (Supplementary Figure S7). The PL lifetime of the Cs-based perovskite films was not measured since the instability of these perovskites in atmospheric conditions (Cs-based perovskites are prone to phase transformation and rapid oxidation in air).

## Conclusions

We investigated the optical, structural, morphology and electronic properties of a broad family of hybrid Sn and Pb iodide compounds with MA, FA and Cs cations stabilised in a 3D perovskite structure. Table 1 summarises the crystallisation, film morphology, lattice change, bandgap range, emission properties and Stark shift of all of the mixed Sn-Pb halide perovskites. In comparison with the MA- and FA-based perovskites, the Cs-based perovskites exhibit a larger bandgap change and wider PL spectrum coverage; however, owing to their low phase stability, it is difficult to fabricate efficient LEDs using the Cs-based perovskites. The properties of the FA- and MA-based perovskites were comparable, and these materials were superior to the Cs-based perovskites in terms of PL, crystallisation and stability. Therefore, MA- and FA-based mixed Sn-Pb halide perovskites are promising candidates for manufacturing low-cost, efficient NIR-LEDs. Moreover, we conclude that Cs-based mixed Sn-Pb halide perovskites do not exhibit an obvious bowing effect due to the absence of organic cations.

**TABLE 1:** Summary of the material and spectral properties of the mixed Sn-Pb halide perovskites.

	MASn_x_Pb_1-x_I_3_	FASn_x_Pb_1-x_I_3_	CsSn_x_Pb_1-x_I_3_
Crystallinity	High	Low	Medium
Film morphology	High coverage	High coverage	Poor coverage
Change in lattice	Small	Small	Large
Bandgap range	1.2–1.55 eV	1.2–1.5 eV	1.3–1.7 eV
Absorption intensity	High	High	High
Emission range	766–980 nm	800–965 nm	716–960 nm
Emission intensity	High	High	Low
PL lifetime	Short	Short	—
Stark shift	Large	Large	Small

## Experimental Section

### Precursor Solution Preparation

The MAPbI_3_ precursor solution was prepared by dissolving 0.3 M MAI and 0.3 M PbI_2_ in 630 μL N,N-dimethylmethanamide (DMF) and 70 μL dimethylsulfoxide (DMSO). The MASnI_3_ precursor was prepared by dissolving 0.3 M MAI and 0.3 M SnI_2_ with 10 mol% (2.4 mg) of SnF_2_ in 800 μL DMF and 200 μL DMSO. The mixed Sn-Pb perovskite precursor solution was obtained by mixing the MAPbI_3_ and MASnI_3_ precursor solutions in stoichiometric amounts. The FAPbI_3_ precursor solution was prepared by dissolving 0.3 M FAI and 0.3 M PbI_2_ in 800 μL DMF and 200 μL DMSO. The FASnI_3_ precursor was prepared by dissolving 0.3 M FAI and 0.3 M SnI_2_ with 10 mol% (2.4 mg) of SnF_2_ in 800 μL DMF and 200 μL DMSO. The mixed Sn-Pb perovskite precursor solution was obtained by mixing the FAPbI_3_ and FASnI_3_ precursor solutions in stoichiometric amounts. The CsPbI_3_ precursor solution was prepared by dissolving 0.3 M CsI and 0.3 M PbI_2_ in 900 μL DMF and 100 μL DMSO. The CsSnI_3_ precursor was prepared by dissolving 0.3 M CsI and 0.3 M SnI_2_ with 10 mol% (2.4 mg) of SnF_2_ in 800 μL DMF and 200 μL DMSO. The mixed Sn-Pb perovskite precursor solution was obtained by mixing the CsPbI_3_ and CsSnI_3_ precursor solutions in stoichiometric amounts. Moreover, 50 μL of PEAI with a concentration of 50 mg/ml in DMF was added to all precursor solutions.

### Perovskite Film Preparation

The patterned ITO-coated glass was cleaned through sonication by using a detergent, deionised water, acetone and isopropyl alcohol. The glass was dried at 80°C in a baking oven and subjected to oxygen plasma treatment for 4 min. Subsequently, the perovskite precursor solution was spin-coated onto the ITO substrates. For the MA-based system, the perovskite precursor solutions were spin-coated through a two-step process: at 1000 rpm for the first 10 s, and at 4000 rpm for the next 30 s. In the last 15 s in the second step, an anti-solvent dripping process with chlorobenzene was used in the spin-coating. Subsequently, the perovskite film was annealed at 100°C for 10 min in an N_2_-filled glove box. For the FA-based system, the perovskite precursor solutions were spin-coated through a two-step process: at 1000 rpm for the first 10 s, and at 4000 rpm for the next 40 s. In the last 10 s in the second step, an anti-solvent dripping process with chlorobenzene was implemented. Subsequently, the FAPbI_3_ perovskite film was annealed at 160°C for 30 min, and the FASn_0.2_Pb_0.8_I_3_ film was annealed at 150°C for 30 min. The other films (Sn = 0.4, 0.6, 0.8, 1) were annealed at 100°C for 10 min in an N_2_-filled glove box. For the Cs-based system, the perovskite precursor solutions were spin-coated through a two-step process: first using 1,000 rpm for 10 s and then using 4,000 rpm for 50 s. The anti-solvent dripping process was not used in the spin-coating. The CsPbI_3_ film, CsSnI_3_ film and mixed Sn-Pb perovskite films were annealed at 150°C for 30 min, 60°C for 10 min and 100°C for 10 min, respectively.

### Characterisation

The ITO/PEDOT:PSS/perovskite structures were subjected to an SEM analysis using a JSM-7001F scanning electron microscope. UV–vis absorption spectroscopy was performed using a UH4150 spectrophotometer. XRD was performed on glass/perovskite structures by using a Bruker D8 Advance X-ray diffractometer equipped with a Cu-Kα X-ray tube. UPS data were obtained using a K-ALPHA + XPS spectrometer. PL spectra were obtained using a spectrofluorometer (Perkin-Elmer LS 55). Luminescence spectra were recorded at ambient temperature using an FLS 980 spectrometer (Edinburgh Instruments) equipped with a 450 W xenon lamp. The PL decay lifetimes were measured using a μF900H high-energy microsecond flash lamp as the excitation source.

## Data Availability

The original contributions presented in the study are included in the article/Supplementary Material, further inquiries can be directed to the corresponding authors.

## Supplementary Material

Supplementary Figure S1 XRD patterns of the pristine perovskite films.

Supplementary Figure S2 XRD patterns of the perovskite films with a PEAI concentration of 100 mg/mL.

Supplementary Figure S3 Tolerance factor for different compositions.

Supplementary Figure S4 SEM images of the perovskite films with different Sn:Pb ratios.

Supplementary Figure S5 A zoomed-in view of the XRD patterns of the peak at around 14°.

Supplementary Figure S6 UPS spectra of MA-based, FA-based and Cs-based perovskites. To obtain the UPS measurements, a He I source with a photon energy of 21.22 eV was used to excite the sample.

Supplementary Figure S7 PL spectrum for each perovskite film composition without normalisation.

Supplementary Figure S8 Stark shift as a function of the Sn:Pb ratio.

Supplementary Table S1 Tolerance factors of perovskite materials with different Sn:Pb ratios.

Supplementary Table S2 Positions of the PL emission peaks of the perovskite films with different Sn:Pb ratios (nm).

## References

[B1] Borek C. Hanson K. Djurovich P. I. Thompson M. E. Aznavour K. Bau R. (2007). Highly Efficient, Near-Infrared Electrophosphorescence from a Pt-Metalloporphyrin Complex. Angew. Chem. Int. Ed. 46, 1109–1112. 10.1002/anie.200604240 17211905

[B2] Cao Y. Wang N. Tian H. Guo J. Wei Y. Chen H. (2018). Perovskite Light-Emitting Diodes Based on Spontaneously Formed Submicrometre-Scale Structures. Nature 562, 249–253. 10.1038/s41586-018-0576-2 30305742

[B3] Chen Z. Li Z. Chen Z. Xia R. Zou G. Chu L.(2021). Utilization of Trapped Optical Modes for White Perovskite Light-Emitting Diodes with Efficiency over 12. Joule 5, 456–466. 10.1016/j.joule.2020.12.008

[B4] Chiba T. Hayashi Y. Ebe H. Hoshi K. Sato J. Sato S. (2018). Anion-exchange Red Perovskite Quantum Dots with Ammonium Iodine Salts for Highly Efficient Light-Emitting Devices. Nat. Phot. 12, 681–687. 10.1038/s41566-018-0260-y

[B5] Deschler F. Price M. Pathak S. Klintberg L. E. Jarausch D.-D. Higler R. (2014). High Photoluminescence Efficiency and Optically Pumped Lasing in Solution-Processed Mixed Halide Perovskite Semiconductors. J. Phys. Chem. Lett. 5, 1421–1426. 10.1021/jz5005285 26269988

[B6] Dimakis E. Jahn U. Ramsteiner M. Tahraoui A. Grandal J. Kong X. (2014). Coaxial Multishell (In, Ga) As/GaAs Nanowires for Near-Infrared Emission on Si Substrates. Nano Lett. 14, 2604–2609. 10.1021/n1500428v10.1021/nl500428v 24678901

[B7] Eperon G. E. Leijtens T. Bush K. A. Prasanna R. Green T. Wang J. T.-W. (2016). Perovskite-perovskite Tandem Photovoltaics with Optimized Band Gaps. Science 354, 861–865. 10.1126/science.aaf9717 27856902

[B8] Fu Y. Wu T. Wang J. Zhai J. Shearer M. J. Zhao Y. (2017). Stabilization of the Metastable Lead Iodide Perovskite Phase *via* Surface Functionalization. Nano Lett. 17, 4405–4414. 10.1021/acs.nanolett.7b01500 28595016

[B9] Goyal A. McKechnie S. Pashov D. Tumas W. van Schilfgaarde M. Stevanović V. (2018). Origin of Pronounced Nonlinear Band Gap Behavior in Lead-Tin Hybrid Perovskite Alloys. Chem. Mat. 30, 3920–3928. 10.1021/acs.chemmater.8b01695

[B10] Gu Y. Guo Z. Yuan W. Kong M. Liu Y. Liu Y. (2019). High-sensitivity Imaging of Time-Domain Near-Infrared Light Transducer. Nat. Photonics 13, 525–531. 10.1038/s41566-019-0437-z

[B11] Hao F. Stoumpos C. C. Chang R. P. H. Kanatzidis M. G. (2014). Anomalous Band Gap Behavior in Mixed Sn and Pb Perovskites Enables Broadening of Absorption Spectrum in Solar Cells. J. Am. Chem. Soc. 136, 8094–8099. 10.1021/ja5033259 24823301

[B12] Hinds S. Myrskog S. Levina L. Koleilat G. Yang J. Kelley S. O. (2007). NIR-emitting Colloidal Quantum Dots Having 26% Luminescence Quantum Yield in Buffer Solution. J. Am. Chem. Soc. 129, 7218–7219. 10.1021/ja070525s 17503821

[B13] Hong W.-L. Huang Y.-C. Chang C.-Y. Zhang Z.-C. Tsai H.-R. Chang N.-Y. (2016). Efficient Low-Temperature Solution-Processed Lead-free Perovskite Infrared Light-Emitting Diodes. Adv. Mat. 28, 8029–8036. 10.1002/adma.201601024 27376676

[B14] Hu M. Chen M. Guo P. ZhouDeng H. J. Deng J. Yao Y. (2020). Sub-1.4eV Bandgap Inorganic Perovskite Solar Cells with Long-Term Stability. Nat. Commun. 11, 151. 10.1038/s41467-019-13908-6 31919343 PMC6952449

[B15] Jiang X. Wang F. Wei Q. Li H. Shang Y. Zhou W. (2020). Ultra-high Open-Circuit Voltage of Tin Perovskite Solar Cells *via* an Electron Transporting Layer Design. Nat. Commun. 11, 1245. 10.1038/s41467-020-15078-2 32144245 PMC7060347

[B16] Kato T. Susawa H. Hirotani M. Saka T. Ohashi Y. Shichi E. (1991). GaAs/GaAlAs Surface Emitting IR LED with Bragg Reflector Grown by MOCVD. J. Cryst. Growth 107, 832–835. 10.1016/0022-0248(91)90565-m

[B17] Kieslich G. Sun S. Cheetham A. K. (2015). An Extended Tolerance Factor Approach for Organic-Inorganic Perovskites. Chem. Sci. 6, 3430–3433. 10.1039/c5sc00961h 28706705 PMC5492664

[B18] Kim D.-H. D’Aléo A. Chen X.-K. Sandanayaka A. D. S. Yao D. Zhao L. (2018). High-efficiency Electroluminescence and Amplified Spontaneous Emission from a Thermally Activated Delayed Fluorescent Near-Infrared Emitter. Nat. Phot. 12, 98–104. 10.1038/s41566-017-0087-y

[B19] Kim H. J. Konarov A. Jo J. H. Choi J. U. Ihm K. Lee H. K. (2019). Controlled Oxygen Redox for Excellent Power Capability in Layered Sodium‐Based Compounds. Adv. Energy Mat. 9, 1901181. 10.1002/aenm.201901181

[B20] Kuo M.-Y. Spitha N. Hautzinger M. P. Hsieh P.-L. Li J. Pan D. (2021). Distinct Carrier Transport Properties across Horizontally vs Vertically Oriented Heterostructures of 2D/3D Perovskites. J. Am. Chem. Soc. 143, 4969–4978. 10.1021/jacs.0c10000 33764051

[B21] Lai M. L. Tay T. Y. S. Sadhanala A. Dutton S. E. Li G. Friend R. H. (2016). Tunable Near-Infrared Luminescence in Tin Halide Perovskite Devices. J. Phys. Chem. Lett. 7, 2653–2658. 10.1021/acs.jpclett.6b01047 27336412

[B22] Leijtens T. Prasanna R. Gold-Parker A. Toney M. F. McGehee M. D. (2017). Mechanism of Tin Oxidation and Stabilization by Lead Substitution in Tin Halide Perovskites. ACS Energy Lett. 2, 2159–2165. 10.1021/acsenergylett.7b00636

[B23] Li Z. Chen Z. Yang Y. Xue Q. Yip H. L. Cao Y. (2019). Modulation of Recombination Zone Position for Quasi-Two-Dimensional Blue Perovskite Light-Emitting Diodes with Efficiency Exceeding 5. Nat. Commun. 10, 1027. 10.1038/s41467-019-09011-5 30833581 PMC6399279

[B24] Lim E. L. Hagfeldt A. Bi D. (2021). Toward Highly Efficient and Stable Sn2+ and Mixed Pb2+/Sn2+ Based Halide Perovskite Solar Cells through Device Engineering. Energy Environ. Sci. 14, 3256–3300. 10.1039/d0ee03368e

[B25] Lin J. T. Liao C. Liao C. C. Hsu C. S. Chen D. G. Chen H. M. (2019a). Harnessing Dielectric Confinement on Tin Perovskites to Achieve Emission Quantum Yield up to 21. J. Am. Chem. Soc. 141, 10324–10330. 10.1021/jacs.9b03148 31244186

[B26] Lin K. Xing J. Quan L. N. de Arquer F. P. G. Gong X. Lu J. (2018). Perovskite Light-Emitting Diodes with External Quantum Efficiency Exceeding 20 Per Cent. Nature 562, 245–248. 10.1038/s41586-018-0575-3 30305741

[B27] Lin R. Xiao K. Qin Z. Han Q. Zhang C. Wei M. (2019b). Monolithic All-Perovskite Tandem Solar Cells with 24.8% Efficiency Exploiting Comproportionation to Suppress Sn(ii) Oxidation in Precursor Ink. Nat. Energy 4, 864–873. 10.1038/s41560-019-0466-3

[B28] Lin R. Xu J. Wei M. Wang Y. Qin Z. Liu Z. (2022). All-perovskite Tandem Solar Cells with Improved Grain Surface Passivation. Nature 603, 73–78. 10.1038/s41586-021-04372-8 35038717

[B29] Liu M. Chen Z. Xue Q. Cheung S. H. So S. K. Yip H.-L. (2018). High Performance Low-Bandgap Perovskite Solar Cells Based on a High-Quality Mixed Sn-Pb Perovskite Film Prepared by Vacuum-Assisted Thermal Annealing. J. Mat. Chem. A 6, 16347–16354. 10.1039/c8ta05444d

[B30] Liu X.-K. Xu W. Bai S. Jin Y. Wang J. Friend R. H. (2021). Metal Halide Perovskites for Light-Emitting Diodes. Nat. Mat. 20, 10–21. 10.1038/s41563-020-0784-7 32929252

[B31] Lu J. Guan X. Li Y. Lin K. Feng W. Zhao Y. (2021). Dendritic CsSnI 3 for Efficient and Flexible Near‐Infrared Perovskite Light‐Emitting Diodes. Adv. Mater. 33, 2104414. 10.1002/adma.202104414 34532897

[B32] NREL (2022). Best Research-Cell Efficiencies. Available at: https://www.nrel.gov/pv/assets/pdfs/best-research-cell-efficiencies-rev220126.pdf (Accessed 26 January 2022).

[B33] Park J. S. Kim S. Xie Z. Walsh A. (2018). Point Defect Engineering in Thin-Film Solar Cells. Nat. Rev. Mat. 3, 194–210. 10.1038/s41578-018-0026-7

[B34] Pazoki M. Jacobsson T. J. Kullgren J. Johansson E. M. J. Hagfeldt A. Boschloo G. (2017). Photoinduced Stark Effects and Mechanism of Ion Displacement in Perovskite Solar Cell Materials. ACS Nano 11, 2823–2834. 10.1021/acsnano.6b07916 28240871

[B35] Qiu W. Xiao Z. Roh K. Noel N. K. Shapiro A. Heremans P. (2019). Mixed Lead-Tin Halide Perovskites for Efficient and Wavelength‐Tunable Near‐Infrared Light‐Emitting Diodes. Adv. Mat. 31, 1806105. 10.1002/adma.201806105 30484911

[B36] Roiati V. Mosconi E. Listorti A. Colella S. Gigli G. De Angelis F. (2014). Stark Effect in perovskite/TiO2 Solar Cells: Evidence of Local Interfacial Order. Nano Lett. 14, 2168–2174. 10.1021/nl500544c 24635762

[B37] Saka T. Hirotani M. Kato T. Susawa H. Yamauchi N. (1993). Bragg Reflector of GaAlAs/AlAs Layers with Wide Bandwidth Applicable to Light Emitting Diodes. J. Appl. Phys. 73, 380–383. 10.1063/1.353860

[B38] Sandanayaka A. S. D. Matsushima T. Adachi C. (2015). Degradation Mechanisms of Organic Light-Emitting Diodes Based on Thermally Activated Delayed Fluorescence Molecules. J. Phys. Chem. C 119, 23845–23851. 10.1021/acs.jpcc.5b07084

[B39] Savill K. J. Ulatowski A. M. Herz L. M. (2021). Optoelectronic Properties of Tin-Lead Halide Perovskites. ACS Energy Lett. 6, 2413–2426. 10.1021/acsenergylett.1c00776 34307880 PMC8291762

[B40] Shi T. Zhang H.-S. Meng W. Teng Q. Liu M. Yang X. (2017). Effects of Organic Cations on the Defect Physics of Tin Halide Perovskites. J. Mat. Chem. A 5, 15124–15129. 10.1039/c7ta02662e

[B41] Shirasaki Y. Supran G. J. Bawendi M. G. Bulović V. (2013). Emergence of Colloidal Quantum-Dot Light-Emitting Technologies. Nat. Phot. 7, 13–23. 10.1038/nphoton.2012.328

[B42] Smith A. M. Mancini M. C. Nie S. (2009). Second Window for *In Vivo* Imaging. Nat. Nanotech 4, 710–711. 10.1038/nnano.2009.326 PMC2862008 19898521

[B43] Song Z. Zhao J. Liu Q. (2019). Luminescent Perovskites: Recent Advances in Theory and Experiments. Inorg. Chem. Front. 6, 2969–3011. 10.1039/C9QI00777F

[B44] Supran G. J. Song K. W. Hwang G. W. Correa R. E. Scherer J. Dauler E. A. (2015). High-performance Shortwave-Infrared Light-Emitting Devices Using Core-Shell (PbS-CdS) Colloidal Quantum Dots. Adv. Mat. 27, 1437–1442. 10.1002/adma.201404636 25639896

[B45] Sutherland B. R. Sargent E. H. (2016). Perovskite Photonic Sources. Nat. Phot. 10, 295–302. 10.1038/nphoton.2016.62

[B46] Tessler N. Medvedev V. Kazes M. Kan S. Banin U. (2002). Efficient Near-Infrared Polymer Nanocrystal Light-Emitting Diodes. Science 295, 1506–1508. 10.1126/science.1068153 11859189

[B47] Wang H.-C. Wang W. Tang A.-C. Tsai H.-Y. Bao Z. Ihara T. (2017). High-Performance CsPb1−x Sn X Br3 Perovskite Quantum Dots for Light-Emitting Diodes. Angew. Chem. Int. Ed. 56, 13650–13654. 10.1002/anie.201706860 28865137

[B48] Wang Q. Wang X. Yang Z. Zhou N. Deng Y. Zhao J. (2019). Efficient Sky-Blue Perovskite Light-Emitting Diodes *via* Photoluminescence Enhancement. Nat. Commun. 10, 5633. 10.1038/s41467-019-13580-w 31822670 PMC6904584

[B49] Wang Z. Wang F. Zhao B. Qu S. Hayat T. Alsaedi A. (2020). Efficient Two-Dimensional Tin Halide Perovskite Light-Emitting Diodes *via* a Spacer Cation Substitution Strategy. J. Phys. Chem. Lett. 11, 1120–1127. 10.1021/acs.jpclett.9b03565 31967834

[B50] Xia Y. Chen Y. Luo T. Liang H. Gao Y. Xu X. (2020). Unexpected Bowing Band Evolution in an All-Inorganic CsSn1−xPbxBr3 Perovskite. RSC Adv. 10, 26407–26413. 10.1039/d0ra03709e 35519736 PMC9055386

[B51] Xiang H. Cheng J. Ma X. Zhou X. Chruma J. J. (2013). Near-infrared Phosphorescence: Materials and Applications. Chem. Soc. Rev. 42, 6128–6185. 10.1039/c3cs60029g 23652863

[B52] Yang Z. Yu Z. Wei H. Xiao X. Ni Z. Chen B. (2019). Enhancing Electron Diffusion Length in Narrow-Bandgap Perovskites for Efficient Monolithic Perovskite Tandem Solar Cells. Nat. Commun. 10, 4498. 10.1038/s41467-019-12513-x 31582749 PMC6776504

[B53] Yu Z. Chen X. Harvey S. P. Ni Z. Chen B. Chen S. (2022). Gradient Doping in Sn-Pb Perovskites by Barium Ions for Efficient Single‐Junction and Tandem Solar Cells. Adv. Mater. 34, 2110351. 10.1002/adma.202110351 35174560

[B54] Zhang X. Wang C. Zhang Y. Zhang X. Wang S. Lu M. (2018). Bright Orange Electroluminescence from Lead-free Two-Dimensional Perovskites. ACS Energy Lett. 4, 242–248. 10.1021/acsenergylett.8b02239

[B55] Zhang Z. Ji R. Kroll M. Hofstetter Y. J. Jia X. Becker‐Koch D. (2021). Efficient Thermally Evaporated γ‐CsPbI 3 Perovskite Solar Cells. Adv. Energy Mat. 11, 2100299. 10.1002/aenm.202100299

[B56] Zhong Y. Dai H. (2020). A Mini-Review on Rare-Earth Down-Conversion Nanoparticles for NIR-II Imaging of Biological Systems. Nano Res. 13, 1281–1294. 10.1007/s12274-020-2721-0 34336144 PMC8323785

[B57] Zhu T. Yang Y. Gong X. (2020). Recent Advancements and Challenges for Low-Toxicity Perovskite Materials. ACS Appl. Mat. Interfaces 12, 26776–26811. 10.1021/acsami.0c02575 32432455

