# Supplementary material for: Wavelength-Tuneable Near-Infrared Luminescence in Mixed Tin–Lead Halide Perovskites
Source: Front Chem. 2022 May 31;10:887983. doi: 10.3389/fchem.2022.887983 (PMC9194474; doi:10.3389/fchem.2022.887983)
Supplement: Supplementary file 1 [file DataSheet1.docx]

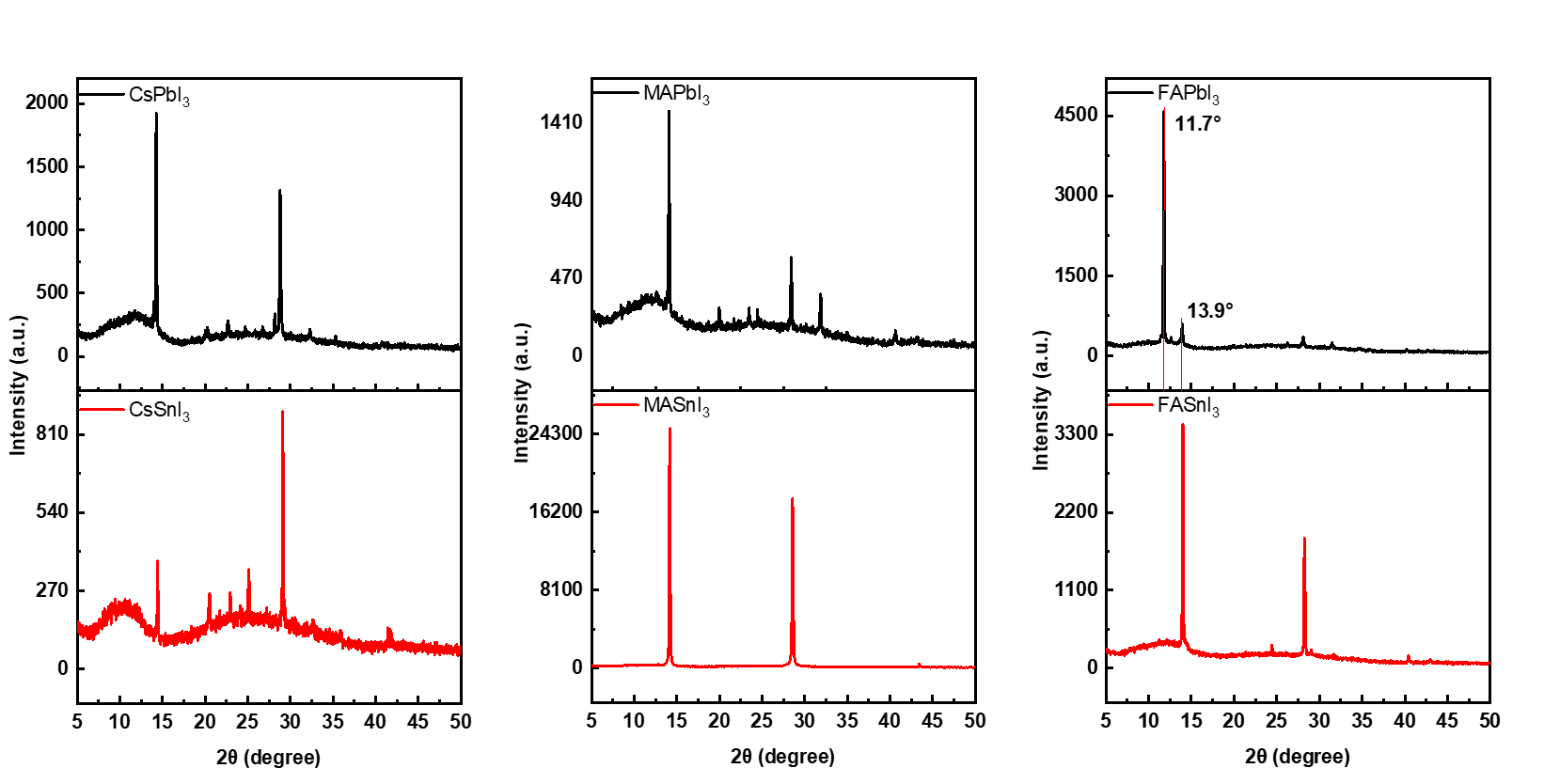


**Figure S1**. XRD patterns of the pristine perovskite films.


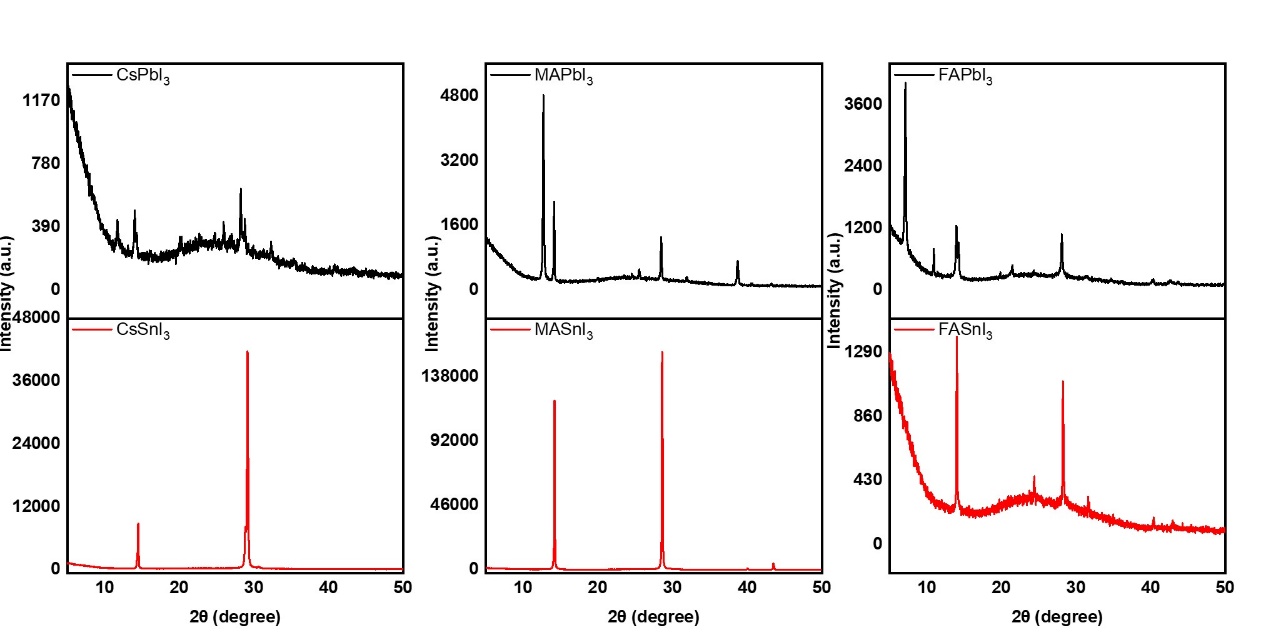


**Figure S2**. XRD patterns of the perovskite films with a PEAI concentration of 100 mg/mL.

**Figure S3**. Tolerance factor for different compositions.

Table S1 Tolerance factors of perovskite materials with different Sn:Pb ratios

| Sn:Pb | 0:1 | 2:8 | 4:6 | 6:4 | 8:2 | 1:0 |
| --- | --- | --- | --- | --- | --- | --- |
| MASn_x_Pb_1-x_I_3_ | 0.909 | 0.911 | 0.914 | 0.915 | 0.918 | 0.92 |
| FASn_x_Pb_1-x_I_3_ | 0.987 | 0.989 | 0.991 | 0.994 | 0.996 | 0.998 |
| CsSn_x_Pb_1-x_I_3_ | 0.807 | 0.809 | 0.811 | 0.813 | 0.815 | 0.817 |


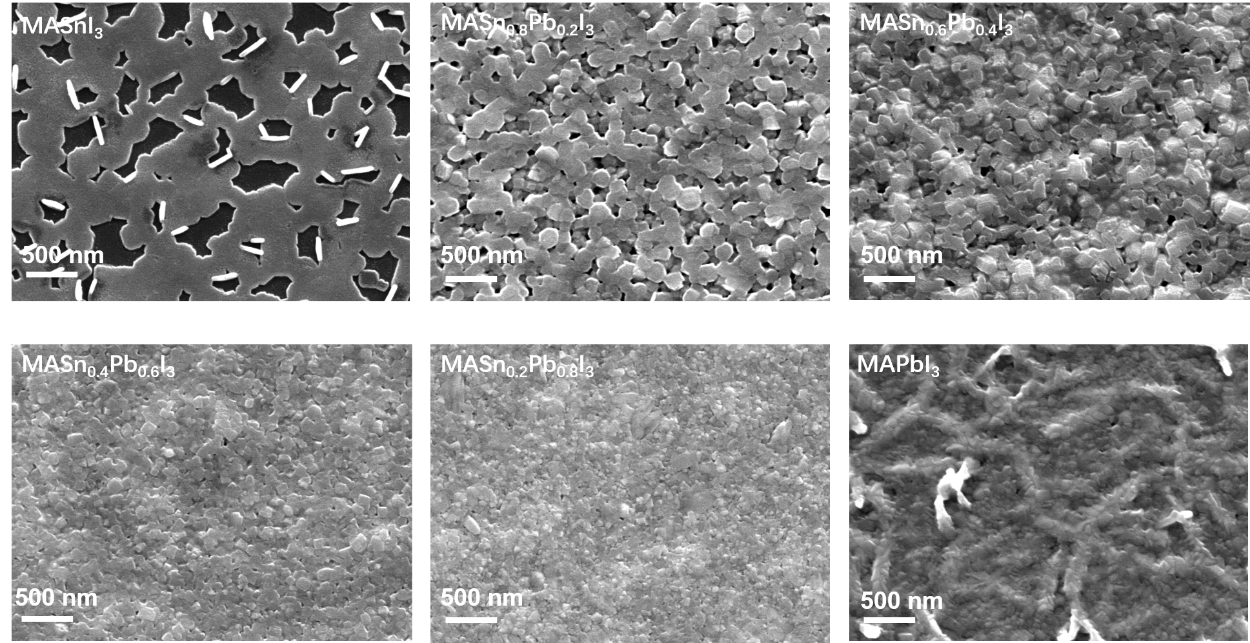


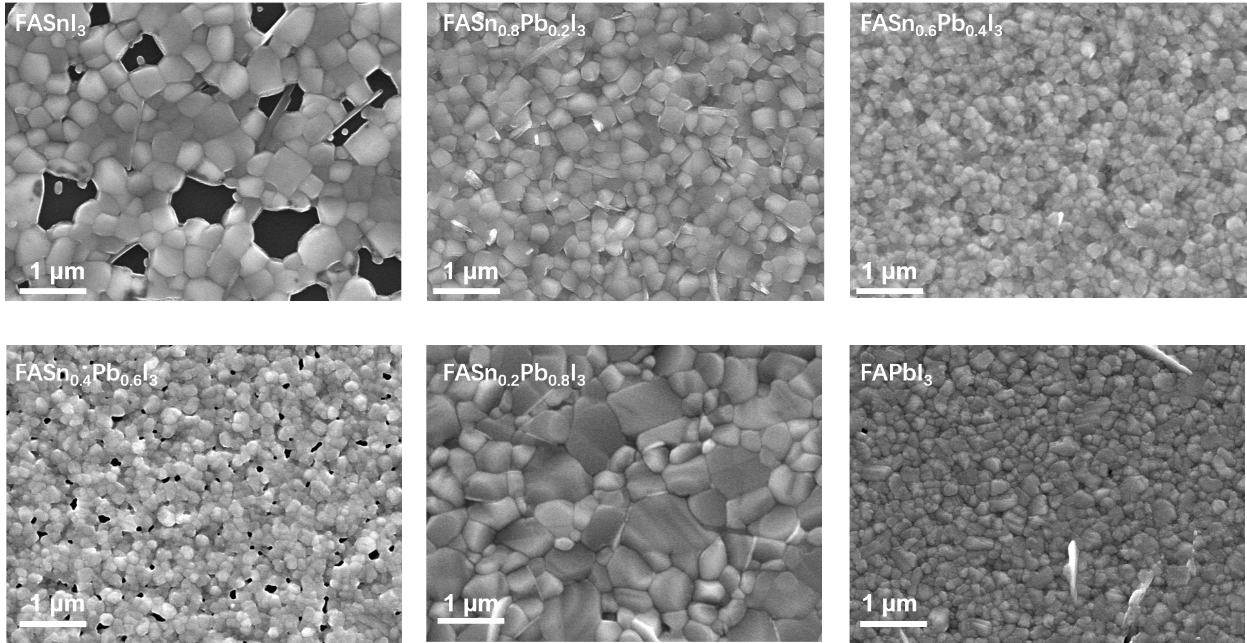


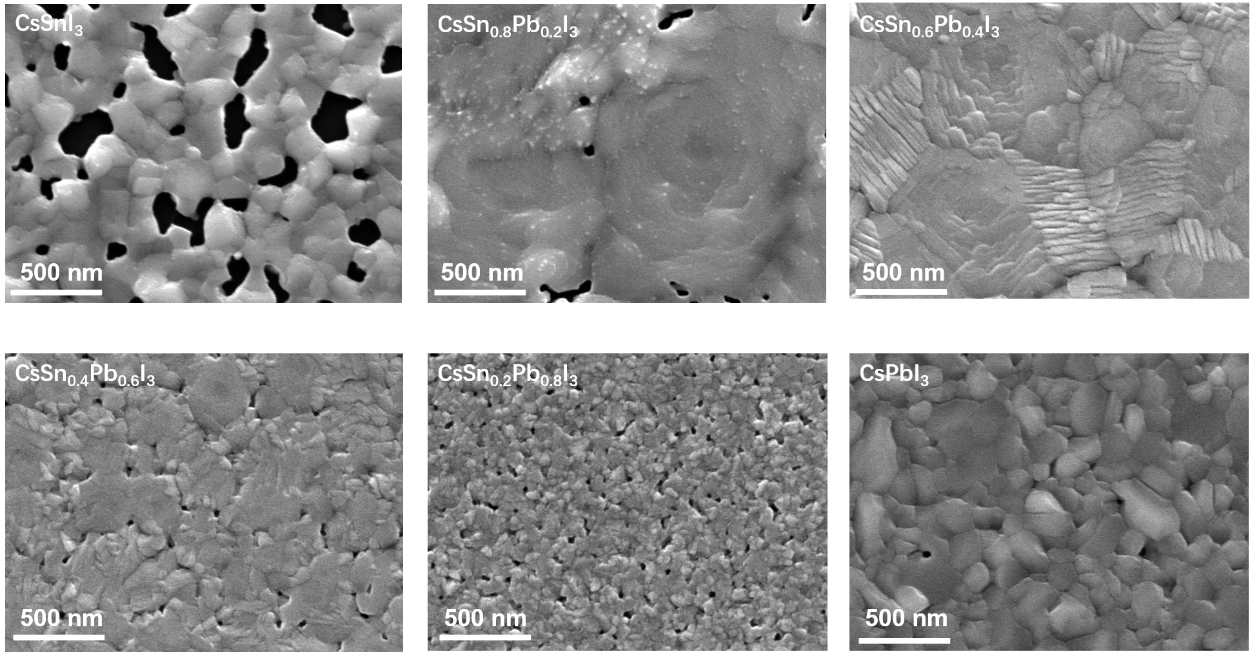


**Figure S4**. SEM images of the perovskite films with different Sn:Pb ratios.

**Figure S5**. A zoomed-in view of the XRD patterns of the peak at around 14°.

**Figure S6**. UPS spectra of MA-based, FA-based and Cs-based perovskites. To obtain the UPS measurements, a He I source with a photon energy of 21.22 eV was used to excite the sample.

**Table S2**. Positions of the PL emission peaks of the perovskite films with different Sn:Pb ratios (nm).

| Sn:Pb | 0:1 | 2:8 | 4:6 | 6:4 | 8:2 | 1:0 |
| --- | --- | --- | --- | --- | --- | --- |
| MASn_x_Pb_1-x_I_3_ | 766 | 884 | 969 | 974 | 982 | 960 |
| FASn_x_Pb_1-x_I_3_ | 800 | 815 | 956 | 963 | 958 | 881 |
| CsSn_x_Pb_1-x_I_3_ | 716 | 870 | 889 | 942 | 961 | 945 |

**Figure S7**. PL spectrum for each perovskite film composition without normalisation.

**Figure S8**. Stark shift as a function of the Sn:Pb ratio.
